# Supplementary material for: Infectious, Allergic, and Immune-Mediated Disease Data Resources: a Landscape Overview and Subset Assessment
Source: J Med Syst. 2025 Nov 22;49(1):169. doi: 10.1007/s10916-025-02302-z (PMC12640313; doi:10.1007/s10916-025-02302-z)
Supplement: Supplementary file 2 — Supplementary Material 2 (DOCX 73.4 KB) [file 10916_2025_2302_MOESM2_ESM.docx]

**Supplementary Table 1.** Data resources and associated URLs identified from publicly available websites and in consultation with National Institute of Allergy and Infectious Diseases-affiliated subject matter experts (n=303).

| Name | URL |
| --- | --- |
| 1000 Genomes Project | <https://www.internationalgenome.org/data> |
| 3D Reconstruction via Stereoscopy for the Study of Mosquito Swarms | <https://www.niaid.nih.gov/research/3d-reconstruction-stereoscopy-study-mosquito-swarms> |
| 4D Nucleome (4DN) | <https://www.4dnucleome.org/> |
| AccessClinicalData@NIAID | <https://www.niaid.nih.gov/research/access-clinical-data-niaid> |
| ACTG/IMPAACT Specimen Repository | <http://www.specimenrepository.org/RepositorySite/home.html> |
| AddGene | <https://www.addgene.org/> |
| AIDS Clinical Trials Group (ACTG) | <https://actgnetwork.org/> |
| ALEdb | <https://aledb.org/> |
| Allele Frequency Net Database | <http://www.allelefrequencies.net/> |
| Allen Institute Human Immune System Explorer | <https://explore.allenimmunology.org/> |
| Alliance of Genome Resources | <https://www.alliancegenome.org/> |
| AllofUs Research Program | <https://allofus.nih.gov/> |
| AmoebaDB | <https://amoebadb.org/amoeba/app> |
| AntibodyRegistry | <https://www.antibodyregistry.org/> |
| ArrayExpress | <https://www.ebi.ac.uk/biostudies/arrayexpress> |
| Aspergillus Genome Database | <https://mycocosm.jgi.doe.gov/Aspnid1/Aspnid1.home.html> |
| aspredicted.org | <https://aspredicted.org/> |
| BacDive | <https://bacdive.dsmz.de/> |
| Bacterial and Viral Bioinformatics Resource Center (BV-BRC) | <https://www.bv-brc.org/> |
| BEIResources | <https://www.beiresources.org/> |
| BibTri v3.0 | <https://bibtri.cepave.edu.ar/webbibtri.php?lang=en> |
| bio.tools | <https://bio.tools/> |
| Bioconductor | <https://www.bioconductor.org/> |
| BioContainers | <https://biocontainers.pro/> |
| Biocyc | <https://biocyc.org/> |
| Bioinformatics and Computational Biosciences Branch Services | <https://www.niaid.nih.gov/research/bcbb-services> |
| Biological General Repository for Interaction Datasets (BioGRID) | <https://thebiogrid.org/> |
| BioModels | <https://www.ebi.ac.uk/biomodels/> |
| Bioregistry | <https://bioregistry.io/> |
| CardioVascular Research Grid | hthttps://www.cvrgrid.org/ |
| Cell Collective | [https://cellcollective.org/#](https://cellcollective.org/) |
| Cell Image Library | <http://www.cellimagelibrary.org/> |
| Cellosaurus | <https://www.cellosaurus.org/> |
| Center for International Blood & Marrow Transplant Research | <https://www.cibmtr.org/Pages/index.aspx> |
| Center for Open Science Pre-registration | <https://www.cos.io/initiatives/prereg> |
| Center for Viral Systems Biology (CViSB) | <https://cvisb.org/> |
| Centers for Research in Emerging Infectious Diseases (CREID) | <https://creid-network.org/> |
| Centers of Excellence for Influenza Research and Response (CEIRR) | <https://www.ceirr-network.org/> |
| ChemDB HIV, Opportunistic Infection and Tuberculosis Therapeutics Database | <https://chemdb.niaid.nih.gov/> |
| ChemokineDB | <https://bioinformatics.niaid.nih.gov/chemokinedb/> |
| Chicago Center for Functional Annotation (CCFA) | <https://www.bv-brc.org/webpage/website/data_collections/content/ccfa.html> |
| CiteAb | <https://www.citeab.com/> |
| ClinEpiDB | <https://clinepidb.org/ce/app> |
| ClinicalGenomeResource (ClinGen) | <https://www.clinicalgenome.org/> |
| ClinicalTrials.gov | <https://clinicaltrials.gov/> |
| ClinRegs | <https://clinregs.niaid.nih.gov/> |
| ClinVar | <https://www.ncbi.nlm.nih.gov/clinvar/intro/> |
| Code Ocean | <https://codeocean.com/> |
| Columbia Lyme Disease Specimen Bank | <https://www.columbia-lyme.org/columbia-specimen-bank> |
| Comprehensive R Archive Network | <https://cran.r-project.org/> |
| Cooperative Centers on Human Immunology (CCHI) | <https://www.niaid.nih.gov/research/cooperative-centers-human-immunology> |
| Coronavirus Resources for Researchers | <https://www.niaid.nih.gov/diseases-conditions/coronavirus-resources> |
| COVID RADx Data Hub | <https://radx-hub.nih.gov/home> |
| COVID-19 Research Database | <https://covid19researchdatabase.org/> |
| CryptoDB | <https://cryptodb.org/cryptodb/app> |
| DAIDS Regulatory Support Center (RSC) | <https://www.niaid.nih.gov/research/daids-regulatory-support-center> |
| Data and Specimen Hub (DASH) | <https://dash.nichd.nih.gov/> |
| Data Discovery Engine-registered Datasets | <https://discovery.biothings.io/dataset?guide=/guide/niaid> |
| Database of Antimicrobial Activity and Structure of Peptides (DBAASP) | <https://dbaasp.org/home> |
| Database of Genotypes and Phenotypes (dbGaP) | <https://www.ncbi.nlm.nih.gov/gap/> |
| Database of Mutations Causing Human Hyper IgE Syndrome (STAT3base) | <https://www.niaid.nih.gov/research/stat3base> |
| Database of Short Genetic Variations (dbSNP) | <https://www.ncbi.nlm.nih.gov/snp/> |
| DisProt database | <https://disprot.org/> |
| Distributed Archives for Neurophysiology Data Integration (DANDI) | <https://dandiarchive.org/> |
| Dockstore | <https://dockstore.org/> |
| Dryad | <https://datadryad.org/> |
| EcoCyc | <https://ecocyc.org/> |
| eDGAR | <http://edgar.biocomp.unibo.it/gene_disease_db/> |
| Electron Microscopy Data Bank (EMDB) | <https://www.ebi.ac.uk/emdb/> |
| ENCODE Project | <https://www.encodeproject.org/about/data-access/> |
| Ensembl | <https://useast.ensembl.org/index.html> |
| European Genome-Phenome Archive (EGA) | <https://ega-archive.org/> |
| European Nucleotide Archive (ENA) | <https://www.ebi.ac.uk/ena/browser/> |
| European Variation Archive (EVA) | <https://www.ebi.ac.uk/eva/> |
| ExpressionAtlas | <https://www.ebi.ac.uk/gxa/home> |
| Extracellular RNA Communication (ExRNA) Atlas | <https://exrna-atlas.org/> |
| FAIRDOMHub | <https://fairdomhub.org/> |
| figshare | <https://figshare.com/> |
| Filariasis Research Reagent Resource Center (FR3) | <http://www.filariasiscenter.org/> |
| FlowRepository | <https://flowrepository.org/public_experiment_representations> |
| Flu Hub | <https://www.fluhub.org/> |
| FlyBase | <https://flybase.org/> |
| Functional Lists of Unknown TB Entities (FLUTE) | in bv brc |
| FungiDB | <https://fungidb.org/fungidb/app> |
| Gemma | <https://gemma.msl.ubc.ca/expressionExperiment/showAllExpressionExperiments.html> |
| GenBank | <https://www.ncbi.nlm.nih.gov/genbank/> |
| Gencode | <https://www.gencodegenes.org/> |
| Gene Expression Omnibus (GEO) | <https://www.ncbi.nlm.nih.gov/geo/> |
| Gene Ontology | <http://geneontology.org/> |
| GeneNetwork | <https://uswest.ensembl.org/index.html> |
| Generalized Proteomics Data Meta-analysis Database (GPMDB) | <https://gpmdb.thegpm.org/> |
| Genes Unknown in Acinetobacter baumannii (GUNK) | <https://www.bv-brc.org/webpage/website/data_collections/content/gunk.html> |
| GenitoUrinary Development Molecular Anatomy Project (GUDMAP) | <https://www.atlas-d2k.org/gudmap/> |
| Genomic Centers for Infectious Diseases (GCID) Resources | <https://www.niaid.nih.gov/research/gcid-resources> |
| Genotype-Tissue Expression (GTEx) Portal | <https://gtexportal.org/home> |
| GiardiaDB | <https://giardiadb.org/giardiadb/app> |
| GISAID | <https://gisaid.org/> |
| GitHub | <https://github.com/> |
| Global Natural Products Social Molecular Networking (GNPS) | <https://gnps.ucsd.edu/ProteoSAFe/static/gnps-splash.jsp> |
| Global Vector Hub | <https://globalvectorhub.lshtm.ac.uk/> |
| GlyGen | <https://data.glygen.org/> |
| gnomAD | <https://gnomad.broadinstitute.org/> |
| GWAS Catalog | <https://www.ebi.ac.uk/gwas/> |
| H3Africa | <https://www.h3abionet.org/resources/h3africa-archive> |
| Harvard DataVerse | <https://dataverse.harvard.edu/> |
| Hemorrhagic Fever Viruses (HFV) Database Project | <https://hfv.lanl.gov/content/index> |
| Hepatitis C Virus Database Project (HCV) | <https://hcv.lanl.gov/content/index> |
| Heterogeneity in Human Immune Cells | <https://heterogeneity.niaid.nih.gov/> |
| HIV Databases | <https://www.hiv.lanl.gov/content/index> |
| HIV Prevention Trials Network (HPTN) | <https://www.hptn.org/> |
| HIV Vaccine Trials Network (HVTN) | <https://www.hvtn.org/> |
| HostDB | <https://hostdb.org/hostdb/app> |
| HuBMap | <https://hubmapconsortium.org/> |
| HUGO Gene Nomenclature Committee (HGNC) | <https://www.genenames.org/> |
| Human Cell Atlas | <https://www.humancellatlas.org/> |
| Human Immunology Project Consortium | <https://www.immuneprofiling.org/hipc/page/show> |
| Human Microbiome Project Portal | <https://portal.hmpdacc.org/projects/t> |
| ICGC Data Portal | <https://dcc.icgc.org/> |
| Illuminating the Drugable Genome (IDG) | <https://druggablegenome.net/> |
| Immcantation Portal | <https://immcantation.readthedocs.io/en/stable/> |
| ImmPort | <https://www.immport.org/shared/home> |
| Immune Epitope Database (IEDB) | <https://www.iedb.org/> |
| immuneAccess | <https://clients.adaptivebiotech.com/immuneaccess> |
| ImmuneSpace | <https://immunespace.org/> |
| ImmuneXpresso | <http://immuneexpresso.org/immport-immunexpresso/public/immunexpresso/search> |
| Immuno Polymorphism Database (IPD) | <https://www.ebi.ac.uk/ipd/> |
| Immunological Genome Project | <https://www.immgen.org/> |
| Immunophenotyping Assessment in COVID-19 Cohort (IMPACC) | <https://docs.immport.org/home/impaccslides/> |
| iModulonDB | <https://imodulondb.org/> |
| INCLUDE Data Coordination Center | <https://includedcc.org/> |
| IndraDB | <https://db.indra.bio/> |
| Infectious Diseases Data Observatory (IDDO) | <https://www.iddo.org/> |
| Influenza Research Database | <https://www.fludb.org/> |
| innateDB | <https://www.innatedb.com/> |
| Integrated Analysis Of Multimodal Single-Cell Data | <https://atlas.fredhutch.org/nygc/multimodal-pbmc/> |
| Integrated Human Microbiome Project (iHMP) | <https://hmpdacc.org/ihmp/> |
| International Committee Taxonomy of Viruses (ICTV) | <https://ictv.global/> |
| International epidemiology databases to evaluate AIDS (IeDEA) | <https://www.iedea.org/> |
| International Human Epigenome Consortium (IHEC) | <https://epigenomesportal.ca/ihec/> |
| International Maternal Pediatric Adolescent AIDS Clinical Trials network (IMPAACT) | <https://www.impaactnetwork.org/> |
| InterPro | <https://www.ebi.ac.uk/interpro/> |
| IPD-IMGT/HLA | <https://www.ebi.ac.uk/ipd/imgt/hla/> |
| iReceptor | <https://gateway.ireceptor.org/login> |
| ITN TrialShare | <https://www.immunetolerance.org/researchers/trialshare> |
| JGI Genome Portal | <https://genome.jgi.doe.gov/portal/> |
| JPOST Repository | <https://globe.jpostdb.org/> |
| Kids First | <https://www.notion.so/Studies-and-Access-a5d2f55a8b40461eac5bf32d9483e90f> |
| Knockout Mouse Phenotyping Program (KOMP2) | <https://www.mousephenotype.org/> |
| Library of Integrated Network-based Cellular Signatures (LINCS) | <https://lincsproject.org/> |
| MACS/WIHS Combined Cohort | <https://statepi.jhsph.edu/mwccs/> |
| Malaria Genomic Epidemiology Network (MalariaGEN) | <https://www.malariagen.net/> |
| MassBank Database | <https://massbank.eu/MassBank/Search> |
| MassIVE | <https://massive.ucsd.edu/ProteoSAFe/static/massive.jsp> |
| Mendeley Data | <https://data.mendeley.com/> |
| MetaboLights | <https://www.ebi.ac.uk/metabolights/> |
| MetabolomeExpress | <http://www.metabolome-express.org/> |
| Metabolomics Workbench | <https://www.metabolomicsworkbench.org/> |
| Microbicide Trials Network (MTN) | <https://www.mtnstopshiv.org/> |
| MicrobiomeDB | <https://microbiomedb.org/mbio/app/> |
| MicrosporidiaDB | <https://microsporidiadb.org/micro/app> |
| Molecular Transducers of Physical Activity in Humans (MoTrPAC) | <https://motrpac-data.org/> |
| Mouse Genome Informatics | <https://www.informatics.jax.org/> |
| Mouse Organogenesis Cell Atlas | <https://oncoscape.v3.sttrcancer.org/atlas.gs.washington.edu.mouse.rna/downloads> |
| Mouse Phenome Database | <https://phenome.jax.org/> |
| MTB Network Portal | <http://networks.systemsbiology.net/mtb/> |
| Multicenter AIDS Cohort Study (MACS) Public Data Set | <https://statepi.jhsph.edu/macs/pdt.html> |
| Mycobrowser | <https://mycobrowser.epfl.ch/> |
| MyExperiment.org | <https://myexperiment.org/home> |
| NIDA National Addiction & HIV Data Archive Program | <https://www.icpsr.umich.edu/web/pages/NAHDAP/index.html> |
| National COVID Cohort Collaborative (N3C) | <https://covid.cd2h.org/> |
| National Database for Autism Research | <https://nda.nih.gov/> |
| National Disease Research Interchange | <https://ndriresource.org/> |
| NCBI BioProject | <https://www.ncbi.nlm.nih.gov/bioproject> |
| NCBI BioSample | <https://www.ncbi.nlm.nih.gov/biosample/> |
| NCBI Geo | <https://www.ncbi.nlm.nih.gov/geo/> |
| NCBI Sequence Read Archive (SRA) | <https://www.ncbi.nlm.nih.gov/sra> |
| NCBI Virus | <https://www.ncbi.nlm.nih.gov/labs/virus/vssi/#/> |
| NCI Genomic Data Commons (GDC) | <https://gdc.cancer.gov/> |
| Nematode.net | Nematode.net |
| Nephele | <https://nephele.niaid.nih.gov/> |
| NeuroMorpho.org | NeuroMorpho.org |
| NeuroVault | <https://neurovault.org/> |
| NHLBIConnects | <https://nhlbi-connects.org/data-request> |
| NIAID Bioinformatics Portal | <https://bioinformatics.niaid.nih.gov/> |
| NICHD DASH | <http://dash.nichd.nih.gov/> |
| NIDKK Central Repository | <https://repository.niddk.nih.gov/home/> |
| NIH AIDS Reagent Program; will be deprecated and combined with BEI Resources on January 14, 2024 | <https://www.hivreagentprogram.org/> |
| NIH BioWulf | <https://hpc.nih.gov/> |
| NIH CDE Repository | <https://cde.nlm.nih.gov/home> |
| NIH Common Fund Data Ecosystem | <https://app.nih-cfde.org/> |
| NIH RePORTER | <https://reporter.nih.gov/> |
| NIHFigShare | <https://nih.figshare.com/> |
| NITRC Neuroimaging Data Repository | <https://www.nitrc.org/xnat/> |
| NLM Data Discovery | <https://datadiscovery.nlm.nih.gov/> |
| NODE | <https://www.biosino.org/node/> |
| Nonhuman Primate Radiation Survivor Late Effects Cohort (NHP RSC) | <https://www.niaid.nih.gov/research/nonhuman-primate-radiation-survivor-late-effects-cohort> |
| Nonhuman Primate Reagent Resource | <https://www.nhpreagents.org/> |
| Non-Obese Diabetic (NOD) Mouse BAC Library | <https://www.sanger.ac.uk/collaboration/sequencing-of-idd-regions-in-the-nod-mouse-genome/> |
| NYU Data Catalog | <https://datacatalog.med.nyu.edu/> |
| Observed Antibody Space | <https://opig.stats.ox.ac.uk/webapps/oas/> |
| Omics DI | <https://www.omicsdi.org/> |
| Open Provenance Model for Workflows Repository | <https://www.opmw.org/> |
| Open Science Framework | <https://osf.io/> |
| OpenfMRI.org | <https://openfmri.org/> |
| OpenNeuro | <https://openneuro.org/> |
| Orfeome | <https://www.niaid.nih.gov/research/orfeome> |
| ORGDB | <https://ogrdb.airr-community.org/> |
| OrthoMCL | <https://orthomcl.org/orthomcl/app> |
| Panther Classification System | <http://pantherdb.org/> |
| Papillomavirus Episteme (PaVE) | <https://pave.niaid.nih.gov/> |
| Pathosystems Resource Integration Center (PATRIC) | in bv brc |
| Patient-Reported Outcomes Measurement Information System (PROMIS) | <https://www.healthmeasures.net/resource-center/research-tools/datasets-for-your-research> |
| PAXDB | <https://pax-db.org/> |
| PeptideAtlas | <http://www.peptideatlas.org/> |
| PharmGKB | <https://www.pharmgkb.org/> |
| PhenoDB | <https://phenodb.org/about> |
| PhysioBank | <https://archive.physionet.org/physiobank/> |
| Physiome Model Repo | <https://models.physiomeproject.org/welcome> |
| PiroplasmaDB | <https://piroplasmadb.org/piro/app> |
| PlasmoDB | <https://plasmodb.org/plasmo/app> |
| Polygenic Score Catalog | <https://www.pgscatalog.org/> |
| Predictive Oncology Model and Data Clearinghouse (MoDaC) | <https://modac.cancer.gov/> |
| Primary Immunodeficiency (PI) Diseases Registry | <https://www.niaid.nih.gov/research/primary-immunodeficiency-diseases-registry> |
| Project TYCHO | <https://www.tycho.pitt.edu/> |
| Protein Data Bank | <https://www.rcsb.org/> |
| Proteome Xchange | <https://www.proteomexchange.org/> |
| Proteomics Identifications Database (PRIDE) | <https://www.ebi.ac.uk/pride/markdownpage/searchpridearchive> |
| Public Health Image Library | <https://phil.cdc.gov/> |
| PubMed Central Code Availability Statements | NA |
| PubMed Central Data Availability Statements | NA |
| PubMed Central Supplemental Information | NA |
| Python Package Index | <https://pypi.org/> |
| Qiita | <https://qiita.ucsd.edu/> |
| Quantitative Set Analysis for Gene Expression (QuSAGE) | <https://www.niaid.nih.gov/research/qusage> |
| Rakai Community Cohort Study (RCCS) | <https://www.rhsp.org/research/rccs/rccs-overview> |
| Rat Genome Database | <https://rgd.mcw.edu/> |
| Reactome | <https://reactome.org/> |
| reframeDB | <https://reframedb.org/> |
| Regional Prospective Observational Research in Tuberculosis (RePORT) | <https://reportinternational.org/> |
| Rep-seq dataset Analysis Platform with an Integrated Antibody Database (RAPID) | NA |
| Roadmap Epigenomics Project | <https://www.ncbi.nlm.nih.gov/geo/roadmap/epigenomics/> |
| RRID Portal | <https://scicrunch.org/resources> |
| Saccharomyces Genome Database (SGD) | <https://yeastgenome.org/> |
| Scripps Consortium for HIV/AIDS Vaccine Development (CHAVD) | <https://www.scripps.edu/science-and-medicine/centers-and-institutes/consortium-for-hiv-aids-vaccine-development/#:~:text=The%20mission%20of%20the%20Consortium,%2C%20genomics%2C%20bioinformatics%20and%20proteomics.> |
| Seven Bridges Public Apps Gallery | NA |
| Signaling Pathways Project (SPP) | <http://www.signalingpathways.org/index.jsf> |
| SimTK | <https://simtk.org/> |
| Stanford University HIV Drug Resistance Database (HIVDB) | <https://hivdb.stanford.edu/> |
| Stimulating Peripheral Activity to Relieve Conditions (SPARC) | <https://sparc.science/about> |
| Structural Database of Allergenic Proteins (SDAP) | <https://fermi.utmb.edu/> |
| Structural Genomics Centers for Infectious Diseases: Resources | <https://www.niaid.nih.gov/research/structural-genomics-centers-infectious-diseases-resources> |
| Synapse | <https://www.synapse.org/> |
| Systems Biology Consortium Resources | <https://www.niaid.nih.gov/research/systems-biology-consortium-resources> |
| TB Portals | <https://tbportals.niaid.nih.gov/> |
| Texas Medical Center Genomic Center for Infectious Diseases (GCID) | <https://gcid.research.bcm.edu/overview> |
| The Broad Institute's Genomic Center for Infectious Diseases (GCID) | <https://www.broadinstitute.org/scientific-community/science/projects/gscid/genomic-center-infectious-diseases> |
| The Cancer Genome Characterization Initiative (CGCI) | NA |
| The Cancer Imaging Archive (TCIA) | NA |
| The Dataverse Project | <https://dataverse.org/> |
| The Global Health Observatory | <https://www.who.int/data/gho/data/indicators> |
| The Institute for Genome Sciences at the University of Maryland School of Medicine Genomic Center for Infectious Diseases (GCID) | <https://gcid.igs.umaryland.edu/> |
| The Network Data Exchange (NDEx) | <https://home.ndexbio.org/about-ndex/> |
| The Pan-Cancer Analysis of Whole Genomes (PCAWG) | NA |
| The World Reference Center for Emerging Viruses and Arboviruses | <https://www.utmb.edu/wrceva> |
| Therapeutically Applicable Research to Generate Effective Treatments initiative (TARGET) | <https://www.cancer.gov/ccg/research/genome-sequencing/target#:~:text=The%20Therapeutically%20Applicable%20Research%20to,less%20toxic%20therapies%20for%20children>. |
| Throughput Ranking by Iterative Analysis of Genomic Enrichment (TRIAGE) | <https://www.niaid.nih.gov/research/triage> |
| ToxoDB | <https://toxodb.org/toxo/app> |
| Trans-Omics for Precision Medicine (TOPMed) | <https://topmed.nhlbi.nih.gov/> |
| Treehouse | <https://treehousegenomics.soe.ucsc.edu/public-data/> |
| TrialShare | <https://www.itntrialshare.org/> |
| TrichDB | <https://trichdb.org/trichdb/app> |
| TriTrypDB | <https://tritrypdb.org/tritrypdb/app> |
| Tuberculosis Regualory Network Analysis Tool (TBRNAT) | <https://www.niaid.nih.gov/research/tuberculosis-regulatory-network-analysis-tool> |
| UCSC Genome Browser | <https://genome.ucsc.edu/> |
| UniProt | <https://www.uniprot.org/> |
| United States Immunodeficiency Network (USIDNET) | <https://usidnet.org/> |
| Vaccine Investigation and Online Information Network (VIOLIN) | <https://violinet.org/introduction.php> |
| VDJ Server | <https://vdjserver.org/> |
| VDJbase | <https://vdjbase.org/> |
| VectorBase | <https://vectorbase.org/vectorbase/app> |
| VEuPathDB | <https://veupathdb.org/> |
| Virtual Biorepository Strain Catalog | <https://arlgcatalogue.org/arlgCatalogue/> |
| Virus Pathogen Research (ViPR) | in BV-BRC |
| Vivli | <https://vivli.org/> |
| Wake Forest Primate Studies Core | <https://www.niaid.nih.gov/research/wake-forest-primate-studies-core> |
| Women's Interagency HIV Study (WIHS) Public Dataset | <https://www.niaid.nih.gov/research/womens-interagency-hiv-study> |
| WorkflowHub | <https://workflowhub.eu/> |
| WorldWide Antimalarial Resistance Network (WWARN) | <https://www.iddo.org/wwarn> |
| Worldwide Protein Databank | <https://www.wwpdb.org/> |
| WormBase | [https://wormbase.org//#012-34-5](https://wormbase.org/#012-34-5) |
| Yale Model Database | <https://senselab.med.yale.edu/modeldb/> |
| Yale Protein Expression Database (YPED) | <https://medicine.yale.edu/keck/nida/yped/> |
| Yoda Project | <https://yoda.yale.edu/> |
| Zebrafish Information Network (ZFIN) | <http://zfin.org/> |
| Zenodo | <https://zenodo.org/> |
| AnVIL | <https://anvilproject.org/data/consortia> |
| BioData Catalyst | <https://gen3.biodatacatalyst.nhlbi.nih.gov/> |
| NIH Helping to End Addiction Long-term (HEAL) Initiative Data Portal | <https://healdata.org/portal/discovery> |
| NCATS Biomedical Data Translator | <https://ui.transltr.io/> |
| NCI Cancer Genomics Cloud | <https://www.cancergenomicscloud.org/datasets> |
| NCATS OpenData Portal | <https://opendata.ncats.nih.gov/covid19> |
| Pathoplexus | <https://pathoplexus.org/> |
| mapMECFS | <https://mapmecfs.org/> |

Note: Not all resources listed are data repositories. This list includes data resources of various types (e.g., repositories, dashboards, knowledgebases).
